# Discovery and Characterization of a Distinctive Theaflavin-3-Gallate Isomer from *Camellia ptilophylla* with Potent Anticancer Properties Against Human Colorectal Carcinoma Cells

**DOI:** 10.3390/foods14040604

**Published:** 2025-02-12

**Authors:** Langhua Zhou, Xiong Gao, Qiuyan Huang, Zhongzheng Chen, Yuanyuan Zhang, Fuming Zhu, Bin Li, Xiaorong Lin

**Affiliations:** 1 College of Food Science, Scientific Research Base of Tea Comprehensive Utilization Technology Integration, Ministry of Agriculture and Rural Affairs, South China Agricultural University, Guangzhou 510642, China; langhuazhou@126.com (L.Z.); huangqiuyan_66@163.com (Q.H.); zhongzhengch@scau.edu.cn (Z.C.); zhangyy@scau.edu.cn (Y.Z.); zhu163fuming@163.com (F.Z.); 2 Institute of Food Microstructure, College of Food and Bioengineering, Fujian Polytechnic Normal University, Fuqing 350300, China; gaoxiong881109@163.com; 3 Guangdong Provincial Key Laboratory of Nutraceuticals and Functional Foods, Guangzhou 510642, China

**Keywords:** *Camellia ptilophylla*, theaflavins, stereoisomer, enzymatic synthesis, colon cancer

## Abstract

Theaflavins, as key bioactive compounds of black tea, are garnering increasing attention. However, research predominantly focuses on theaflavin monomers derived from the enzymatic oxidation of *cis*-type catechins. In this study, we identify a unique stereoisomer of theaflavin-3-gallate (TF-3-G), named isoneoTF-3-G, in black tea from *Camellia ptilophylla* (*C. ptilophylla*), which is rich in *trans*-catechins. IsoneoTF-3-G, a characteristic theaflavin of *C. ptilophylla* black tea, is formed by the oxidation of gallocatechin gallate and catechin. It exhibits a bright orange–red color and shows an [M+H]^+^ ion at *m*/*z* 717.1449 in positive electron spray ionization-mass spectrometry. Furthermore, isoneoTF-3-G demonstrates potent inhibitory effects on the proliferation of human colorectal carcinoma HCT116 cells, with a half-inhibitory concentration of 56.32 ± 0.34 μM. This study reveals that the mitochondrial pathway is involved in the apoptosis induction of HCT116 cells by isoneoTF-3-G. Specifically, isoneoTF-3-G leads to increased reactive oxygen species in HCT116 cells, decreased mitochondrial membrane potential, and the consequent release of cytochrome *c* from the mitochondria to the cytosol, activating caspase-9 and caspase-3, which further promotes the cleavage of poly(ADPribose) polymerase. The results of this study enhance our understanding of the composition and synthesis mechanisms of theaflavins and provide foundational evidence for the further development of isoneoTF-3-G and *C. ptilophylla*.

## 1. Introduction

Tea is one of the most widely consumed non-alcoholic beverages worldwide due to its significant health benefits, including antioxidant, anti-obesity, and anti-inflammatory activity. However, the presence of caffeine, a central nervous system stimulant, limits its consumption in caffeine-sensitive individuals, as excessive caffeine intake can lead to side effects such as insomnia and anxiety [1]. Consequently, the development of low-caffeine tea products has become a key research focus. Despite efforts, an efficient, safe, environmentally friendly, and economical method for artificial decaffeination remains elusive.

In 1981, *Camellia ptilophylla* Chang (*C. ptilophylla*), also known as cocoa tea, was discovered in Southern China [2]. This unique tea variety is naturally low in caffeine. Traditionally used as a health beverage and medication [3], cocoa tea has garnered interest for its distribution [4], genetics [5], processing [6], toxicity [7], chemical composition [8], and bioactive effects [3,9,10]. Notably, cocoa tea contains significantly higher levels of *trans*-catechins [9], such as gallocatechin gallate (GCG), gallocatechin (GC), catechin gallate (CG), and catechin (CA). These compounds constitute 11.32–19.02% of dried cocoa tea, compared to only 2.14–4.49% in traditional tea [3,6]. Rare polyphenolic compounds like 1,2,4,6-tetra-O-galloyl-β-D-glucopyranose and gallocatechin-3,5-di-O-gallate [9], as well as a novel proanthocyanidin GC-(4→8)-GCG [10], have also been identified in cocoa tea, contributing to its stronger biological activity [3,10,11,12,13]. However, research has primarily focused on green cocoa tea, leaving the functional potential of black cocoa tea (BCT) unclear.

**Figure 1:**
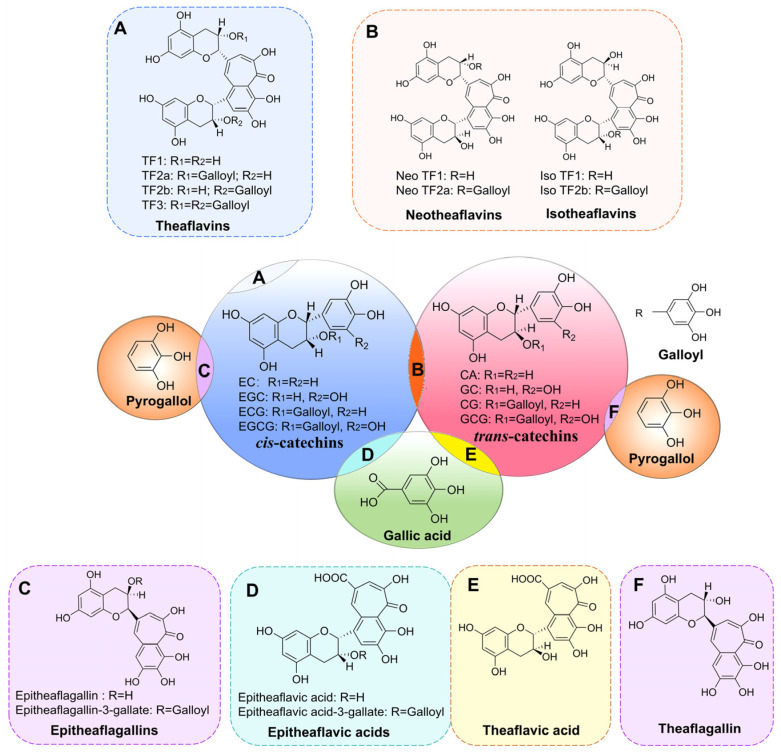
Structures and precursors of major theaflavin derivatives. TF1, theaflavin; TF2a, theaflavin-3-gallate; TF2b, theaflavin-3′-gallate; TF3, theaflavin-3,3′-digallate; Neo TF1, neotheaflavin; Neo TF2a, neotheaflavin-3-gallate; Iso TF1, isotheaflavin; Iso TF2b, isotheaflavin-3′-gallate; EC, epicatechin; EGC, epigallocatechin; ECG, epicatechin gallate; EGCG, epigallocatechin gallate; CA, catechin; GC, gallocatechin; CG, catechin gallate; GCG, gallocatechin gallate.

Theaflavins, characterized by a benzotropolone structure [14], are reddish orange pigments and key components in black tea. They not only influence the flavor and taste of tea [15,16] but also exhibit various biological properties [17], including anticancer [18], antioxidant [19], weight loss [20], anti-aging [21], and antidiabetic [22] effects. Theaflavins are formed through the oxidation of catechins in tea leaves, catalyzed by polyphenol oxidase (PPO), with their chemical structure being significantly influenced by the precursor configuration [23,24]. Four major theaflavin monomers—theaflavin (TF1), theaflavin-3- gallate (TF-3-G, TF2a), theaflavin-3′-gallate (TF-3′-G, TF2b), and theaflavin-3,3′-digallate (TF-3,3′-DG, TF3)—are formed through the co-oxidation of prevalent *cis*-catechins (Figure 1A) [25]. Additionally, there are lower-abundance stereoisomers, such as neotheaflavins and isotheaflavins (Figure 1B), which arise from specific co-oxidation pathways involving both *cis*- and *trans*-catechins [26,27,28,29,30,31]. Other benzotropolone derivatives, such as epitheaflagallins (Figure 1C), epitheaflavic acids (Figure 1D), theaflavic acid (Figure 1E), and theaflagallins (Figure 1F), formed by specific catechins, further increase black tea’s chemical diversity [25,32,33].

The chemical structure of theaflavins in cocoa tea differs from that in traditional tea, primarily due to the unique composition of catechins in cocoa tea, where *trans*-catechins serve as the main precursors for oxidation synthesis under PPO. In our previous study, we found a novel theaflavin in BCT [9], but its structure and health effects are not yet fully understood. In this study, the theaflavin composition in BCT was preliminarily analyzed using ultra-high-performance liquid chromatography–quadrupole-time-of-flight tandem mass spectrometry (UPLC-Q-TOF-MS/MS). The formation mechanisms of theaflavins in BCT were explored by catalyzing different trans-catechin combinations with PPO. Additionally, the inhibitory effects of the highly abundant novel theaflavin, isoneotheaflavin-3-gallate (isoneoTF-3-G), from BCT on human colon cancer (HCT116) cells and its involvement in the mitochondrial apoptosis pathway were investigated. This study expands the understanding of the theaflavin composition and synthesis mechanisms in BCT, providing scientific evidence for its potential application in colorectal cancer therapy.

## 2. Materials and Methods

### 2.1. Materials and Chemical Reagents

BCT was prepared as outlined in our previous study [9]. Fresh cocoa tea leaves were subjected to indoor withering and rolling, followed by fermentation. The fermented leaves underwent an initial drying phase at 110–120 °C for 15 min, followed by secondary drying at 85–95 °C for an additional 15 min. The dried BCT was ground and sieved to obtain particles with a mesh size ranging from 20 to 30. Fresh Fengshui pears were purchased locally and stored at 4 °C until their use on the following day.

(+)-Catechin hydrate (≥98%), CG (≥98%), GC (≥98%), TF1 (≥98%), TF2a (≥98%), TF2b (≥98%), and TF3 (≥98%) were purchased from Shanghai Yuanye Bio-Technology Co., Ltd. (Shanghai, China). Ascorbic acid, disodium hydrogen phosphate dodecahydrate (Na_2_HPO_4_·12H_2_O), and citric acid were purchased from Guangzhou Chemical Reagent Factory (Guangzhou, China). Ethylene diamine tetra acetic acid (EDTA) was obtained from Tianjin Kemiou Chemical Reagent Co., Ltd. (Tianjin, China). Ethyl acetate was obtained from Sinopharm Chemical Reagent Co., Ltd. (Shanghai, China). 5-Fluorouracil (5-FU, 99% biotechnology grade), polyvinylpolypyrrolidone (PVPP), methanol-d4 (D,99.8%) + 0.03% (*v*/*v*) tetramethyl silane (TMS), and trifluoroacetic acid of high-performance liquid chromatography (HPLC) grade were acquired from Shanghai Macklin Biochemical Technology Co., Ltd. (Shanghai, China). HPLC-grade acetonitrile was acquired from Merck (Darmstadt, Germany), and HPLC-grade acetonitrile was obtained from Anpel Laboratory Technologies, Inc. (Shanghai, China). Methyl thiazolyl tetrazolium (MTT), Coomassie brilliant blue G-250 (Sigma-Aldrich, St. Louis, MO, USA), RPMI-1640 medium, fetal bovine serum (FBS, Australia Origin), Dulbecco’s phosphate-buffered saline (pH 7.4), 0.25% trypsin–EDTA (Gibco, Carlsbad, CA, USA), penicillin/streptomycin solution (HyClone, Uppsala, Sweden), a reactive oxygen species (ROS) assay kit, a mitochondrial membrane potential (MMP) assay kit (Beyotime, Haimen, Jiangsu, China), a Pierce bicinchoninic acid (BCA) protein assay kit, an enhanced chemiluminescent substrate (Thermo Scientific, Rockford, IL, USA), 10× Tris-buffered saline tween (TBST) buffer (Sangon Biotech, Shanghai, China), primary antibodies against β-actin, cytochrome *c*, poly (ADPribose) polymerase (PARP), cleaved PARP, horseradish peroxidase-conjugated secondary antibody (Cell Signaling Technology, Boston, MA, USA), caspase-8, and a caspase-9 colorimetric assay kit (KeyGEN BioTECH, Nanjing, China) were also used.

### 2.2. Preparation of BCT Extracts

The extraction method was modified according to the report by Vural et al. [34]. Briefly, 5 g of BCT was mixed with 50 mL of 30% aqueous ethanol and stirred at 70 °C for 25 min. The mixture was filtered, and the residue was re-extracted using the same procedure. Combined filtrates were concentrated under reduced pressure using a rotary evaporator (R-3, BUCHI, Flawil, Switzerland). The concentrate was then partitioned twice with equal volumes of ethyl acetate. The ethyl acetate layers were concentrated, and the solvent was exchanged with ultrapure water. The solution was lyophilized using a freeze dryer (Christ Alpha 1-2 LD plus, Osterode, Germany) to yield BCT powder, which was stored at −20 °C for subsequent analysis.

### 2.3. Preparation of PPO from Pears

PPO was prepared from pears using the methods of Zou et al. [35] and Zhou et al. [36], with appropriate modifications. Fifty grams of fresh Fengshui pear was homogenized with 50 mL of pre-cooled phosphate–citrate buffer (pH 5.5, containing 1.0% PVPP, 0.1% ascorbic acid, 1 mmol/L EDTA, *w*/*v*) in a blender for 1 min, with a half-stop for 1 min on ice. The mixture was then incubated for 12 h at 4 °C. The resulting suspension was filtered through gauze and centrifuged at 8000× *g* for 30 min at 4 °C. The supernatant, containing PPO, was collected.

### 2.4. Co-Oxidation of Trans-Catechins by PPO

Three independent substrate combinations of *trans*-catechins (GCG+CA, GCG+CG, GC+CA) were each dissolved at 6 mmol/L in citric acid–phosphate buffer (pH 5.5). PPO from the pears was added to each substrate mixture to constitute 37% of the total volume. The mixture was incubated at 32 °C for 30 min. After incubation, each mixture was extracted twice with ethyl acetate. The ethyl acetate layers were then concentrated under reduced pressure and nitrogen gas.

### 2.5. HPLC Analysis

Samples were analyzed using a Shimadzu LC 2030C 3D plus HPLC instrument (Shimadzu Corporation, Kyoto, Japan) equipped with an Agilent Eclipse XDB-C18 column (4.6 mm × 250 mm, 5 μm) (Agilent, Santa Clara, CA, USA), following Lin’s method [8], with minor modifications. The mobile phase consisted of 0.05% trifluoroacetic acid in ultrapure water (phase A) and acetonitrile (phase B). A 10 μL sample was injected and separated by gradient elution at 1 mL/min at 40 °C, with the following gradients: 0–5 min, 4–5.5% B; 5–25 min, 5.5–9.5% B; 25–49 min, 9.5–21.5% B; 49–76.5 min, 21.5–27.0% B. Detection was performed at 280 nm, and quantification was based on standards.

### 2.6. Isolation and Purification of BCT Theaflavins

The theaflavins were initially separated using preparative high-performance liquid chromatography (PHPLC) (LC3000, Beijing Innovation Tong Heng Technology Co., Ltd., Beijing, China) coupled with a COSMOSIL 5C18-MS-II column (20 mm I.D × 250 mm, 5 μm) (Nacalai Tesque, Inc., Kyoto, Japan). The mobile phase consisted of water (phase A) and acetonitrile (phase B), with the following elution program: 0–30 min, isocratic elution with 13% B; 30–50 min, 13–22% B; 50–70 min, 22–40% B. The flow rate was maintained at 15 mL/min, the column temperature was set at 25 °C, and the detection wavelength was 210 nm. Further purification was conducted using a Shimadzu LC 2030C 3D plus HPLC system (Shimadzu Corporation, Kyoto, Japan) connected to a ZORBAX Eclipse XDB-C18 semi-preparative column (9.4 × 250 mm, 5 μm) (Agilent, Santa Clara, CA, USA). The mobile phase was composed of 0.5% trifluoroacetic acid (phase A) and acetonitrile (phase B), with the elution program as follows: 0–23 min, isocratic elution with 19.5% acetonitrile; 23–23.1 min, 19.5–20% B; 23.1–32 min, isocratic elution with 20% acetonitrile. The flow rate was set at 4 mL/min, the column temperature was 35 °C, and the detection wavelengths were 210 nm and 280 nm.

### 2.7. Identification of BCT Theaflavins

The theaflavins and PPO-catalyzed oxidation products were identified using a UPLC Q-TOF-LC/MS/MS instrument (1290-6540B, Agilent, Santa Clara, CA, USA) with an ECLIPSE PLUS C18 column (1.8 µm, 2.1 × 100 mm) (Agilent, Santa Clara, CA, USA) and with electron spray ionization (ESI) as the ion source. Full MS scans were conducted in both positive and negative modes from *m*/*z* 100 to 1300 and *m*/*z* 100 to 1100, respectively. The specific parameters for the mass spectrometer were as follows: gas temperature, 300 °C; gas flow, 8 L/min; nebulizer, 45 pigs; sheath gas temperature, 350 °C; sheath gas flow, 10 L/min. Nuclear magnetic resonance (NMR) spectra were recorded on an AVANCE NEO 600 Digital NMR Spectrometer (Bruker, Fällanden, Switzerland) operating at ^1^H and ^13^C resonance frequencies of 600 and 151 MHz, respectively. Chemical shift values were referenced to TMS at 0 ppm. Samples dissolved in methanol were analyzed using an ultraviolet–visible (UV–Vis) spectrophotometer (HITACHIU-2900, Hitachi High, Tokyo, Japan) at wavelengths ranging from 190 to 800 nm, with a scan speed of 400 nm/min.

### 2.8. Anticancer Activity of IsoneoTF-3-G

#### 2.8.1. Cell Culture

Human colon cancer HCT116 and HT29 cells, obtained from the cell bank of the Chinese Academy of Sciences (Shanghai, China), were cultured in RPMI-1640 and DMEM medium, respectively. Both cell lines were cultured in media supplemented with 10% FBS and 1% penicillin/streptomycin and at 37 °C in a 5% CO_2_ atmosphere.

#### 2.8.2. Cell Viability Assay

Cell viability was evaluated using the MTT reduction assay [3]. HCT116 and HT29 cells were seeded at a density of 5.0 × 10^3^ cells per well in a 96-well plate and incubated for 24 h at 37 °C in a 5% CO_2_ atmosphere. Fresh medium containing isoneoTF-3-G (0–210 µM, supplemented with 0.05% DMSO as a solubilizing agent) was added to the wells and incubated for 48 h. 5-FU was used as a positive control. After incubation with the MTT solution for 2 h, formazan crystals were dissolved in DMSO. The plate was then incubated at 37 °C with gentle shaking for 15 min. Subsequently, 150 μL of the solution from each well was transferred to a new 96-well plate. Absorbance was measured at 550 nm using a VersaMax ELISA Microplate Reader (Molecular Devices, Sunnyvale, CA, USA). Cell viability was calculated as a percentage of the control group, and the half-inhibitory concentration (IC_50_) values were determined using the Origin 8.5 software.

#### 2.8.3. Measurement of Intracellular ROS

The ROS levels were evaluated using an ROS assay kit with a 2′,7′-dichlorodihydrofluorescein diacetate (DCFH-DA) fluorescence probe. Cells were seeded at a density of 2.5 × 10^4^ cells per well in a 24-well plate and incubated for 24 h at 37 °C in a 5% CO_2_ atmosphere. Subsequently, the cells were treated with isoneoTF-3-G at concentrations of 0, 50, 100, and 150 µM for 48 h. The cells were then stained with 10 μΜ DCFH-DA for 30 min in the dark. Fluorescence images were captured using a fluorescence microscope (Axio Observer A1, Carl Zeiss, Oberkochen, Germany). The fluorescence intensity of each image was quantified using the ImageJ software (version 1.53k).

#### 2.8.4. MMP Assay

For the assessment of MMP changes in cells, an MMP assay kit incorporating JC-1 was utilized. Cells were initially seeded at a density of 3.0 × 10^5^ cells per well in a 60 mm culture dish and incubated for 24 h at 37 °C under 5% CO_2_. Following this, they were co-incubated with isoneoTF-3-G for 48 h. Subsequently, the cells were collected, stained with JC-1, and analyzed using a SpectraMax i3x fluorescence plate reader (Molecular Devices, Sunnyvale, CA, USA). The JC-1 monomer was detected at an excitation/emission wavelength of 490/530 nm (green fluorescence), while the JC-1 polymer was detected at 525/590 nm (red fluorescence). The red-to-green fluorescence ratio was then calculated to evaluate mitochondrial depolarization.

#### 2.8.5. Activation of Caspase-3 and Caspase-9 Proteins

Cells treated with varying concentrations of isoneoTF-3-G were collected, and lysis buffer was added to obtain the supernatants of the cell lysates. The protein concentration was measured [37] and adjusted to 100–200 μg to ensure consistency. Following the manufacturer’s instructions, 50 μL of the cell lysate supernatant with a consistent protein concentration was pipetted, and different reagents were added sequentially to initiate the reaction. The absorbance was measured at λ = 400 nm using a microplate reader to determine the activation levels of the caspase proteins.

#### 2.8.6. Western Blotting Analysis

Cells treated with isoneoTF-3-G were lysed in a radioimmunoprecipitation assay buffer on ice for 30 min, followed by centrifugation at 14,000× *g* for 15 min at 4 °C. The supernatant was collected, and the protein concentrations were measured using a BCA protein assay kit (Thermo Scientific, Rockford, IL, USA). The protein concentrations were then normalized to ensure consistency. Equal amounts of protein were subjected to SDS–polyacrylamide gel electrophoresis and transferred onto polyvinylidene fluoride membranes (Millipore, Billerica, MA, USA). The membranes were blocked with 5% skim milk in TBST at room temperature for 1 h and incubated with specific primary antibodies overnight at 4 °C and subsequently with horseradish peroxidase-conjugated secondary antibodies for 1 h. Protein bands were visualized using an enhanced chemiluminescent substrate and imaged using the OmegaLum G software (v2.1.1027.0) (Aplegen, San Francisco, CA, USA).

### 2.9. Statistical Analysis

All experiments were performed with three biological replicates. All results are presented as the means ± standard deviation (S.D.). The statistical significance of the differences was assessed by ANOVA followed by the Tukey–Kramer test using the SPSS 20.0 software for Windows. The Origin 8.5 software and ImageJ software (version 1.53k) were used for data visualization and illustration.

## 3. Results and Discussion

### 3.1. Composition of Theaflavins in BCT

**Figure 2:**
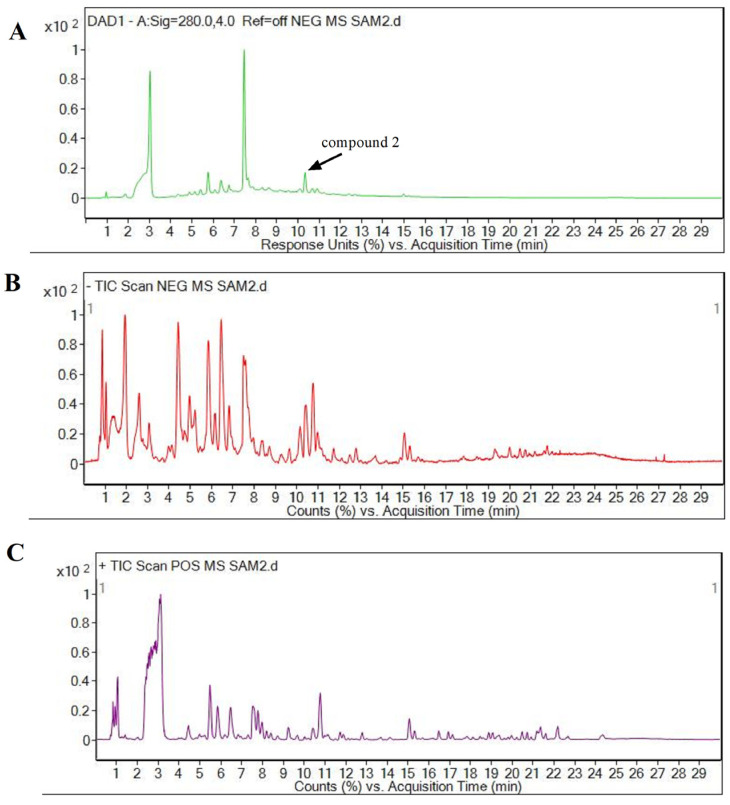
Total ion chromatogram of the MS for black tea ethanol/ethyl acetate extracts from *C. ptilophylla*. (**A**) DAD (green line); (**B**) negative ion mode (red line); (**C**) positive ion mode (purple line).

To investigate the composition of the theaflavins in BCT, alcohol/ethyl acetate extracts were analyzed using electron spray ionization mass spectrometry (ESI-MS) in both positive and negative ion modes (Figure 2). The MS spectra and MS^2^ fragments of the theaflavins are detailed in Table 1**.** The analysis tentatively identified three categories of oxidized products from catechins: stereoisomers of theaflavins (compounds **1**, **2**, **3**, **4**, **5**), theaflavic acids (compounds **6**, **7**, **8**, **9**), and theaflagallins (compounds **10**, **11**).

Compound **1**, identified as isoneotheaflavin, showed molecular ions [M−H]^−^ at *m*/*z* 563.1206 and [M+H]^+^ at *m*/*z* 565.1339. Isotheaflavins maintain the *trans*- and *cis*-configuration of the C-ring of flavan-3-ols, resulting from the coupling of trihydroxy (pyrogallol) at the B-rings of *trans*-catechins and dihydroxy (catechol) at the B-rings of *cis*-catechins [29,30,31]. Similarly, neotheaflavins contain the *cis*- and *trans*-configuration of the C-ring of flavan-3-ols after coupling trihydroxy (pyrogallol) at the B-rings of *cis*-catechins and dihydroxy (catechol) at the B-rings of *trans*-catechins [27,28,38]. The formation of benzotropolone depends on the condensation of di- and tri-hydroxyls at the B-rings of flavan-3-ols, irrespective of the C-ring configuration [26,39]. Given the high content of GC and CA in *C. ptilophylla* [13] and the MS^2^ spectra of compound **1**, it likely includes the double *trans*-configuration of the C-ring of flavan-3-ols, and it probably formed through the co-oxidation of GC and CA [40]. Davies et al. reported a similar theaflavin, termed “B” theaflavin, derived from GC and CA [41], while its exact structure remains unknown, suggesting the potential for similarity or distinction between “B” theaflavin and compound **1**.

Compound **2**, identified as isoneoTF-3-G, showed molecular ions [M−H]^−^ at *m*/*z* 715.1314 and [M+H]^+^ at *m*/*z* 717.1435, as previously proposed by our group [9]. Compounds **3**, **4**, and **5**, with the same ion peaks [M−H]^−^ at *m*/*z* 867.1418 and similar fragment ions at *m*/*z* 169, 389, and 527 but differing retention times, were tentatively identified as theaflavin-3,3′-digallate 1, 2, and 3, respectively, based on their stereoisomers [32].

Theaflavic acids, containing one benzotropolone moiety, result from oxidation between the tri-hydroxyls of gallic acid and B-ring tri-hydroxyls [26]. Previous studies have shown that theaflavic acid, epitheaflavic acid, and epitheaflavic acid 3-gallate can be synthesized enzymatically from the reactions of CA, EC, and ECG with gallic acid [41]. However, due to the low concentrations of precursors like gallic acid and ECG in fresh tea leaves, the formation of theaflavic acids is limited [3,13]. Thus, analytical methods with high sensitivity, high quality, and high accuracy are essential in detecting theaflavic acids in black tea. In this study, compound **6**, with molecular ions [M−H]^−^ at *m*/*z* 427.0674 and [M+H]^+^ at *m*/*z* 429.1508, was identified as theaflavic acid [42,43]. Compounds **7**, **8**, and **9**, with the same [M−H]^−^ ion at *m*/*z* 579.0784 and similar fragment ions at retention times of 8.607, 8.837, and 9.589 min, respectively, were identified as epitheaflavic acid 3-gallate or its isomer, theaflavic acid 3-gallate. This novel theaflavic acid is likely derived from the reaction of GC and gallic acid.

Theaflagallins, characterized by the 1′,2′,3′-trihydroxy-3,4-benzotropolone unit, are produced by the oxidative coupling of the B-ring tri-hydroxyls of flavan-3-ols and 1,2,3-trihydroxybenzene (pyrogallol) or gallic acid [44]. Tan et al. reported that epitheaflagallin could be formed via the auto-oxidation of EGC, even in the absence of pyrogallol or gallic acid [32]. In this study, compound **10**, identified as theaflagallin, showed molecular ions [M−H]^−^ at *m*/*z* 399.0728 and [M+H]^+^ at *m*/*z* 401.0851, based on previous studies [32]. Compound **11**, preliminarily identified as epitheaflagallin 3-O-gallate and previously discovered in black tea [45], or its novel stereoisomer theaflagallin 3-O-gallate, showed ions [M−H]^−^ at *m*/*z* 551.084 and [M+H]^+^ at *m*/*z* 553.0959. These results highlight the unique composition of the theaflavins in BCT, driven by their distinct composition of catechins.

### 3.2. Enzymatic Synthesis of BCT Theaflavins

**Figure 3:**
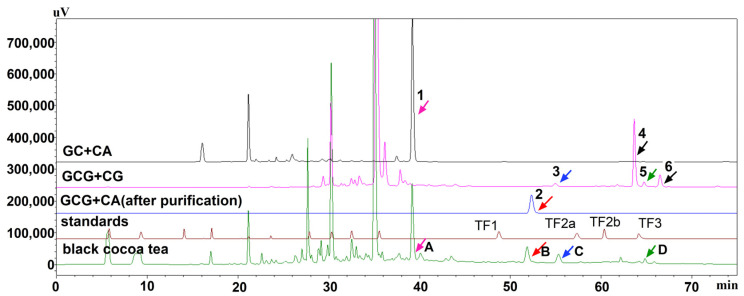
HPLC chromatograms of theaflavins. GC+CA (gray line), GCG+CG (pink line), and GCG+CA (blue line) were used as independent substrate combinations and were catalyzed by PPO to produce different products (**1**–**6**) compared with standards (red line) and BCT (green line). TF1 (theaflavin), TF2a (theaflavin-3-gallate, TF-3-G), TF2b (theaflavin-3′-gallate, TF-3′-G), and TF3 (theaflavin-3,3′-digallate, TF-3,3′-DG) are theaflavin standards. Components A–D were detected in the BCT extract. The retention times of these components match those of products **1**–**6** from the independent substrate combinations. Specifically, component A (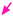) corresponds to product **1** from the GC+CA combination, component B (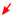) corresponds to product **2** from the GCG+CA combination, and components C (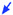) and D (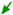) correspond to products **3** and **5** from the GCG+CG combination.

To confirm the structure of the BCT theaflavins, we compared them with four common theaflavin standards. As illustrated in Figure 3, no theaflavins in BCT matched the retention times of these standards, indicating that BCT theaflavins have different structures [9].

To further explore the formation mechanism of BCT theaflavins, we used groups including GC+CA, GCG+CG, and GCG+CA, catalyzed by Fengshui pear PPO. The oxidized products were analyzed using HPLC. In the GC+C group, a peak at 39.203 min, close to compound **A** [9,33], was observed. This suggests that compound **A** was probably a novel theaflavin and formed through the co-oxidation of GC and CA.

In the GCG+CG group, four products were detected. The retention times of product **3** and product **5** were almost identical to those of compounds **C** and **D** of BCT at 54.987 min and 64.852 min, respectively, indicating that these products are also present in BCT. Product **4**, with a high response value, was the main product in the enzymatic synthesis by GCG+CG, followed by product **6**. This suggests that the enzymatic oxidation of GCG+CG produced various products. During oxidation, GCG contributes a pyrogallol B-ring and a galloyl group, while CG provides a catechol B-ring and a galloyl group. Enzymes first oxidize catechol B-rings, forming quinone, which subsequently oxidizes the pyrogallol B-rings due to their lower redox potential compared to catechol rings [25,41]. The higher redox potential of the galloyl group and its lower reactivity with o-quinones result in minor product formation [25], leading to a complex mixture [44].

Our previous study suggested that compound **B** was probably a novel theaflavin (isoneoTF-3-G) in BCT [9]. In this study, the major product **2** from GCG+CA at 52.32 min matched the retention time of isoneoTF-3-G. The differences in the retention time between product **2** and the four common theaflavin standards, as shown in Figure S1 (Supplementary Materials), confirm that product ***2*** has a different chemical structure compared to non-isomeric theaflavins. According to the formation mechanism of theaflavins, the enzymatic oxidation of *trans*-catechin mainly forms theaflavins in the *trans*-conformation. As shown in Figure 3, isoneoTF-3-G, with higher relative content, is considered the dominant theaflavin in BCT.

### 3.3. Identification of Oxidation Products of GCG and C

**Figure 4:**
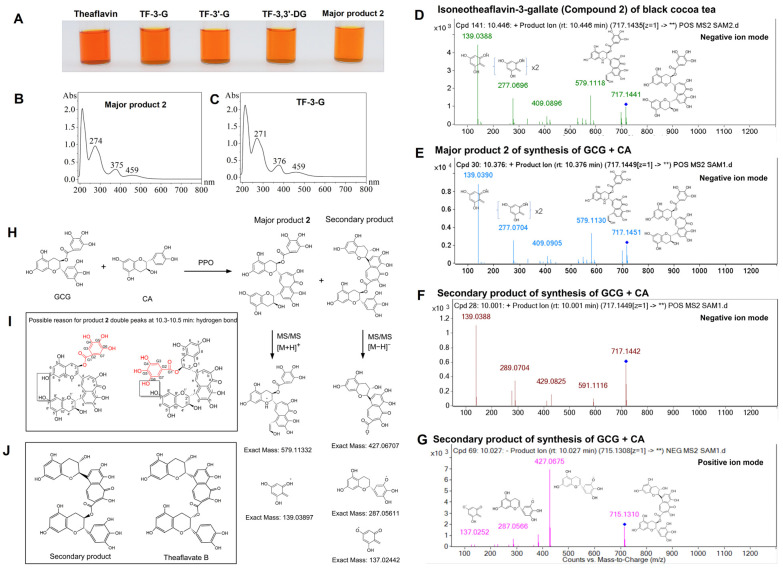
Structural identification of oxidation products of GCG and CA. (**A**) The colors of four major theaflavin standards and product **2** are presented. (**B**) The UV–Vis spectra of product **2** in methanol, with (**C**) TF-3-G as a standard, are shown. (**D**) The ESI-MS^2^ spectra of isoneoTF-3-G from BCT are displayed. (**E**–**G**) The ESI-MS^2^ spectra of major product **2** and the secondary product in the negative/positive ion modes are also depicted. (**H**) A schematic representation of the potential fragment pathways for both the major and secondary products is included. The structures of (**I**) major product **2** and (**J**) the secondary product are illustrated. Major product **2** and the secondary products were derived from the PPO-catalyzed oxidation of GCG and CA.

To confirm the structure of product **2**, formed through the co-oxidation of GCG and C, we conducted combined separation using PHPLC and HPLC with a semi-preparative column. The chemical structure of product **2** was subsequently identified via UV–Vis spectra, ^1^H-NMR, ^13^C-NMR, and Q-TOF-LC/MS/MS. As shown in Figure 4A, product 2 exhibited a brilliant reddish orange color, similar to the other four common theaflavins, indicating that product **2** was one of the theaflavin pigments. The UV–Vis spectra of product **2** showed characteristic absorption peaks at 274, 375, and 459 nm, close to the TF-3-G standard (Figure 4B,C), suggesting similarity in the structures of product **2** and TF-3-G.

The ^1^H and ^13^C NMR spectra and data for product **2** are shown in Figure S2 and Table S1 (Supplementary Materials). The spectra are listed as follows: ^1^H NMR (600 MHz, methanol-d4):δ 7.96 (s, 1H), 7.57 (s, 1H), 7.29 (d, J = 1.3 Hz, 1H), 6.92 (s, 2H), 5.41 (q, J = 6.5 Hz, 1H), 5.27 (s, 1H), 5.09–5.05 (m, 1H), 4.15 (s, 1H), 4.09 (q, J = 7.1 Hz, 2H), 2.97 (dd, J = 16.3, 5.3 Hz, 1H), 2.85 (s, 1H), 2.80 (dd, J = 16.2, 7.2 Hz, 1H), 2.56 (dd, J = 15.9, 8.5 Hz, 1H), 2.04–1.97 (m, 4H), 1.61 (t, J = 6.1 Hz, 2H), 1.48 (q, J = 6.0 Hz, 1H), 1.40 (qd, J = 6.3, 3.4 Hz, 1H), 1.34–1.30 (m, 3H), 1.28 (s, 3H), 1.23 (t, J = 7.1 Hz, 2H), 0.89 (t, J = 6.9 Hz, 0H). ^13^C NMR (151 MHz, Methanol-d4):δ 186.23, 175.37, 173.17, 167.46, 158.38, 157.96, 157.70, 157.63, 156.62, 156.06, 155.85, 151.93, 147.32, 146.52, 140.10, 133.58, 130.57, 121.19, 117.40, 110.23, 100.85, 99.74, 82.50, 70.99, 61.69, 49.71, 33.85, 27.05, 26.78, 24.02, 23.86, 20.99, 20.86, 14.58.

In the ^13^C NMR spectrum, a benzotropolone skeleton was determined through six carbon signals, namely C-a (δ 186.23), C-d (δ133.58), C-f (δ130.57), C-g (δ155.85), C-h (δ151.93), and C-i (δ147.32). The galloyl ester group signals were found at C-G1 (δ 167.46), C-G2 (δ121.19), C-G4 (δ146.52), C-G5 (δ140.1), and C-G6 (δ146.52). According to the NMR data of product **2** and the reference values for theaflavins [31], the molecular structure of product **2**, consisting of a benzotropolone skeleton and two flavan-3-alcohol units, was suggested.

The ESI-LC-MS/MS pattern of product **2** confirmed the molecular formula of C_36_H_28_O_16_, exhibiting characteristic ion peaks and ionic fragments (Table 2 and Figure 4E), similar to isoneoTF-3-G (compound **2**) (Table 1 and Figure 4D). The total ion chromatogram of the MS for product **2** is shown in Figure S3 (Supplementary Materials). According to these results, product **2** was confirmed as the novel stereoisomer of TF-3-G, isoneoTF-3-G (Figure 4). In particular, we found that product **2** showed double peaks at 10.37–10.50 min, with all ion peaks observed as [M+H]^+^ at *m*/*z* 717.1449, featuring the same fragment ions. The double peaks of product **2** might result from the formation of different intramolecular hydrogen bonds induced by bond rotation (Figure 4I).

Notably, the secondary enzymatic oxidation product of GCG and CA by PPO was likely another novel theaflavin. As shown in Table 2 and Figure 4F,G, the secondary product exhibited the same [M+H]^+^ peaks at *m*/*z* 717.1449 and a similar molecular formula (C_36_H_28_O_16_) to isoneoTF-3-G, but with different ionic fragments. We speculate that the secondary product was an isomer of theaflavate B with an additional hydroxyl group (Figure 4J). Peaks with similar MS^2^ fragmentation patterns were generally designated as stereoisomers, while those with different MS^2^ fragmentation patterns were designated as isomers with different positions of atoms or groups [32]. Although the secondary product (Figure 4H) was synthesized by the PPO-catalyzed co-oxidation of GCG and CA, it was not detected in BCT.

These findings confirmed the structure of isoneoTF-3-G as the predominant theaflavin monomer formed through the co-oxidation of GCG and CA in BCT. Several studies have reported the protective effect of theaflavins against colon cancer [18,46,47,48]. However, the bioactivity of isoneoTF-3-G remains unknown. Therefore, we further explored the inhibitory effects of isoneoTF-3-G on the proliferation of human colon cancer cells, using TF-3-G as a control, and also assessed its induction of mitochondrial apoptosis.

### 3.4. Inhibition of HCT116 Cells by IsoneoTF-3-G

#### 3.4.1. Proliferation Inhibitory Activity of IsoneoTF-3-G in HCT116 Cells

**Figure 5:**
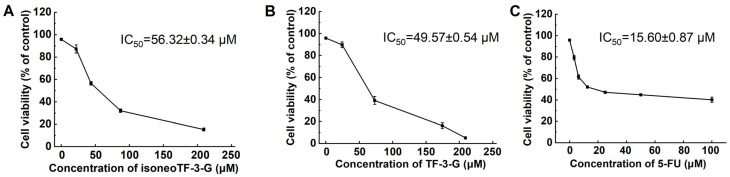
Cytotoxic effects of isoneoTF-3-G, TF-3-G, and 5-FU on HCT116 Cells. MTT assay was used to determine the cytotoxic effects of isoneoTF-3-G (**A**), TF-3-G (**B**), and 5-FU (**C**) on HCT116 cells after 48 h. Viability is expressed as a percentage of the control (100%). A concentration of 0 μM indicates cells treated with the sample solvent only (negative control, cell viability > 95%). 5-FU was used as the positive control. Data are shown as means ± S.D. (*n* = 3).

Theaflavins have been demonstrated to induce cell apoptosis across a range of cancer cell lines [18,46,47,48]. In our study, we investigated the inhibitory effects of isoneoTF-3-G and TF-3-G on two human colon cancer cell lines, HCT116 and HT29, with 5-FU serving as a positive control. The results indicated that isoneoTF-3-G, TF-3-G, and 5-FU exerted potent inhibitory effects on HCT116 cells, with IC_50_ values of 56.32 ± 0.34 μM, 49.57 ± 0.54 μM, and 15.60 ± 0.87 μM, respectively (Figure 5). However, these compounds did not exhibit significant inhibitory effects on HT29 cells, with isoneoTF-3-G and TF-3-G showing no inhibitory effects even at concentrations up to 200 μM (Figure S4). The differential sensitivity of HCT116 and HT29 cells to isoneoTF-3-G and TF-3-G suggests that these compounds may target specific pathways or mechanisms that are differentially expressed or active in these cell lines. Imran et al. [49] have reported that theaflavins significantly inhibit the proliferation of HCT116 cells by inducing apoptosis and cell cycle arrest in a dose- and time-dependent manner. In contrast, HT29 cells may possess distinct anti-apoptotic pathways that protect them from theaflavin-induced cell death [50]. The differential responses of HCT116 and HT29 cells to theaflavins underscore the importance of elucidating the molecular mechanisms underlying the anticancer effects of these compounds. This knowledge could facilitate the development of more targeted and effective preventive and therapeutic strategies for colon cancer using theaflavins.

Different structural forms of theaflavins exhibit varying anticancer capabilities, which are closely related to their chemical structures, particularly the substitution positions of galloyl groups [51]. Tan et al. [18] showed that theaflavins containing galloyl groups, such as TF-3-G, TF-3′-G, and TF-3,3′-DG, exhibit higher cytotoxicity against human colon cancer cells (HCT116), indicating that the substitution of galloyl groups in theaflavins enhances their anticancer activity. Moreover, TF-3-G and TF-3′-G both contain two galloyl groups, but their different connection positions also affect their anticancer activity. Our study also found that isoneoTF-3-G and TF-3-G, which are isomers of each other, showed differences in their inhibitory effects on HCT116 cells due to the differences in their connection sites. This suggests that isomers of compounds may have different efficacies, which is related to the structure–activity relationship of the compounds, and these mechanisms are worth further investigation.

Therefore, further understanding the mechanisms by which isoneoTF-3-G, a novel theaflavin derived from *C. ptilophylla*, inhibits the proliferation of HCT116 cells is of significant importance for its development. This knowledge could contribute to the development of more targeted and effective preventive and therapeutic strategies for colon cancer using theaflavins.

#### 3.4.2. Induction of Intracellular ROS in HCT116 Cells by IsoneoTF-3-G

**Figure 6:**
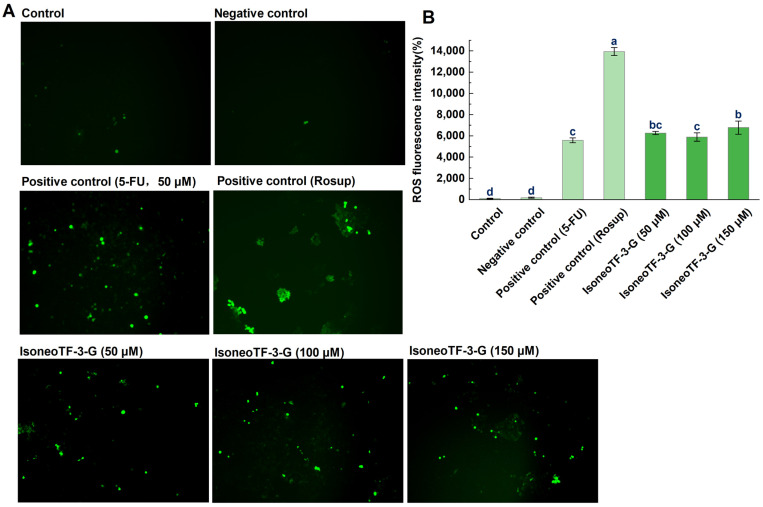
Effects of isoneoTF-3-G on ROS generation in HCT116 cells. (**A**) Images of DCFH-DA-stained HCT116 cells (100× magnification). (**B**) The fluorescence intensity of each image was quantified using the ImageJ software (version 1.53k). Cells were treated with isoneoTF-3-G at concentrations of 50, 100, and 150 µM for 48 h. The negative control indicates cells treated with the sample solvent only. Rosup from the ROS assay kit and 5-FU were used as positive controls. Data are presented as mean ± standard deviation (*n* = 3), with different letters indicating significant differences (*p* < 0.05).

Previous studies indicate that a slight increase in intracellular ROS promotes cell proliferation and differentiation, while a significant surge induces cell apoptosis [52,53]. Many chemotherapy drugs and natural compounds achieve therapeutic effects by inducing ROS accumulation in HCT116 cells [54,55]. In this study, we measured the intracellular ROS levels using a DCFH-DA fluorescence probe to assess the effects of isoneoTF-3-G on ROS accumulation in HCT116 cells. As shown in Figure 6, the DCF fluorescence intensity increased in a dose-dependent manner in the isoneoTF-3-G-treated cells compared to the control group, indicating significant intracellular ROS accumulation triggered by isoneoTF-3-G treatment. This ROS production contributes to the apoptosis of cancer cells [56], as observed by Maity et al., who reported that theaflavin-conjugated gold nanoparticles induced apoptosis in human ovarian teratoma cells (PA-1) by simulating ROS generation [57]. Notably, after 48 h of treatment with isoneoTF-3-G, sustained ROS levels were still detectable in the HCT116 cells. This indicates that isoneoTF-3-G can maintain a prolonged state of oxidative stress in these cells, and the mitochondrial apoptotic program may also be activated. We further investigated the potential apoptotic pathways induced by isoneoTF-3-G in HCT116 cells.

#### 3.4.3. Activation of Mitochondrial Apoptosis in HCT116 Cells by IsoneoTF-3-G

**Figure 7:**
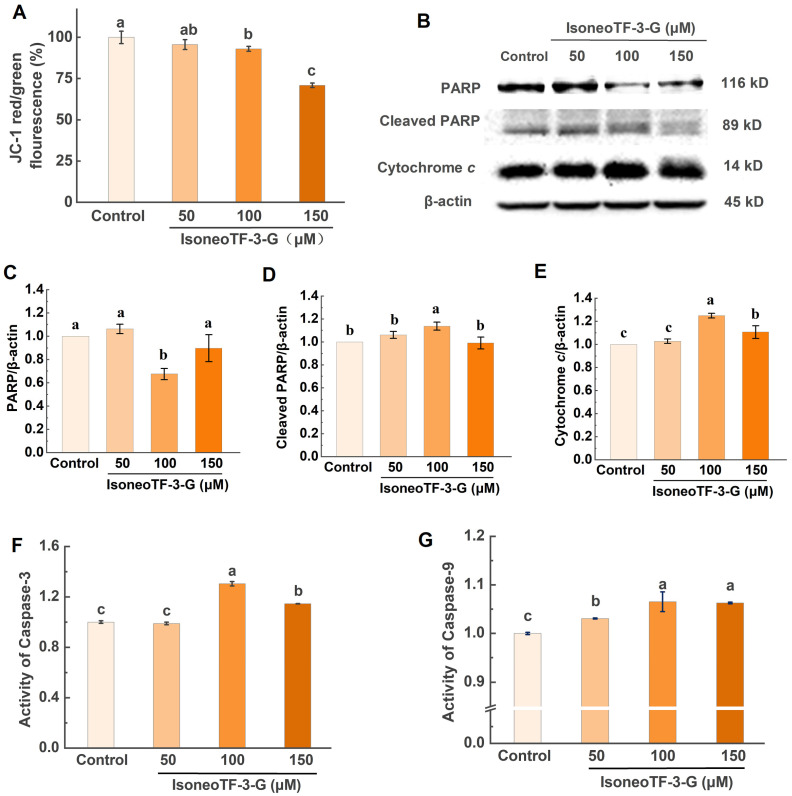
Activation of the mitochondrial apoptotic pathway in isoneoTF-3-G-treated HCT116 cells. (**A**) Effects of isoneoTF-3-G on intracellular MMP in HCT116 cells. Cells stained with JC-1 were analyzed using a fluorescence plate reader. The relative ratio of red and green fluorescence was used to measure the degree of mitochondrial depolarization. The protein bands (**B**) and (**C**–**E**) expression levels of PARP, cleaved PARP, and cytochrome *c* in HCT116 cells were detected by Western blot analysis. The relative density of the bands was normalized with β-actin. The activation degree of the caspase-3 protein (**F**) and caspase-9 protein (**G**) was analyzed by caspase colorimetric assay kits. The data are presented as mean values ± S.D. (*n* = 3). Different letters in the same title indicate significant differences (*p* < 0.05) among different treatment groups.

Mitochondria play a crucial role in cell apoptosis. A decrease in MMP is considered an early event in the apoptosis cascade [58,59]. To further explore the inhibitory effects of isoneoTF-3-G on HCT116 cell proliferation, we measured the MMP changes using an MMP assay kit with JC-1. Figure 7A shows a significant decrease in MMP in HCT116 cells treated with high concentrations of isoneoTF-3-G. The marked reduction in the red/green fluorescence intensity ratio indicates mitochondrial damage. The effective concentration of isoneoTF-3-G to reduce the MMP in HCT116 at 100 μM (71.6 μg/mL) is lower than that of green tea polyphenols, such as GTP (200 μg/mL) [60] and EGCG (100 μg/mL [61], 200 μM [62]), which require higher concentrations to achieve similar effects.

Once the cell death signal is identified, the MMP decreases, making the mitochondrial outer membrane permeable. This permeability leads to the release of cytochrome *c*, endonuclease G, and other intermembrane space proteins into the cytosol, activating downstream caspases and initiating apoptosis [58,63]. Caspase-9, an initiator caspase in the mitochondria-dependent pathway, activates caspase-3, a key executor of caspases in apoptosis [3,64]. We investigated the involvement of the mitochondrial apoptotic pathway in the anticancer activity of isoneoTF-3-G by detecting the expression of related proteins. Figure 7B–G shows a significant increase in the cytoplasmic cytochrome *c* levels and upregulated caspase-3 and caspase-9 activity in the isoneoTF-3-G-treated HCT116 cells compared to the control. Additionally, there was a notable decrease in PARP protein expression and a significant increase in the cleaved PARP protein levels, confirming that isoneoTF-3-G induces apoptosis in HCT116 cells through the mitochondrial pathway. This finding aligns with a previous report that TF1 reduces HeLa cell proliferation and induces mitochondrial apoptosis by increasing cytochrome *c* release, caspase-3 and -9 activation, and the cleaved PARP levels [56].

These findings indicate that isoneoTF-3-G exhibits similar inhibitory effects on human colon cancer cells to TF-3-G, with the mitochondrial apoptosis pathway playing a significant role in its mechanism. Given the complexity of cellular regulatory systems, future studies should explore additional regulatory pathways involved in the anticancer activity of isoneoTF-3-G.

## 4. Conclusions

This study confirmed the existence and formation mechanism of a novel isoneoTF-3-G in the black tea of *C. ptilophylla*, as well as its strong inhibitory effect on the proliferation of human colorectal carcinoma cells (HCT116). Unlike traditional tea, the theaflavins in *C. ptilophylla* black tea are not dominated by the four conventional theaflavins but by the polymerization of *trans*-catechins, forming isoneoTF-G-3, a stereoisomer of TF-G-3. IsoneoTF-G-3, characterized by its brilliant reddish orange color, is formed from the co-oxidation of GCG and C and shows [M+H]^+^ ions at *m*/*z* 717.1449 in positive ESI-MS. This study revealed that the mitochondrial pathway was involved in the apoptosis induction of HCT116 cells by isoneoTF-3-G. This compound promotes intracellular ROS accumulation and reduces the cellular MMP, leading to the release of cytochrome *c* from the mitochondria into the cytoplasm. The elevated levels of cytochrome *c*, along with activated caspase-9 and caspase-3, further promote the cleavage of PARP. These findings suggest that isoneoTF-3-G’s antiproliferative activity in HCT116 cells could be harnessed for colon cancer prevention. Overall, this research provides fundamental insights for the potential development of isoneoTF-3-G as a natural active ingredient in *C. ptilophylla* for colorectal cancer prevention.

## Figures and Tables

**Table 1:** The MS spectra and MS^2^ fragments of BCT theaflavins by UPLC/Q-TOF-MS/MS in the negative/positive ion modes.

Compound	RT (min) Negative/ Positive Ion Modes	MS (*m*/*z*) [M−H]^−^/[M+H]^+^	Formula	MS^2^ (*m*/*z*) [M−H]^−^/[M+H]^+^	Tentative Identification
**1**	8.734/8.717	563.1206/565.1339	C_29_H_24_O_12_	125.0249, 137.0239, 241.0510, 425.0863/ 139.0388, 277.0690, 427.1016, 565.1333	Isoneotheaflavin
**2**	10.437/10.446	715.1314/717.1435	C_36_H_28_O_16_	125.0239, 169.0136, 321.0761, 545.1098, 563.1188/ 139.0388, 277.0696, 409.0896, 579.1118	Isoneotheaflavin-3-gallate
**3**	11.088/-	867.1418/-	C_43_H_32_O_20_	169.0155, 389.0685, 527.0982, 545.1110, 715.1254/-	Theaflavin-3,3′-digallate 1
**4**	11.354/-	867.1418/-	C_43_H_32_O_20_	169.0142, 389.0689, 527.0964, 545.1075, 697.1161, 715.1343/-	Theaflavin-3,3′-digallate 2
**5**	12.481/12.523	867.1418/869.1546	C_43_H_32_O_20_	125.0238, 169.0143, 389.0664, 527.0977, 697.1220/ 139.0395, 277.0689, 391.0807, 529.1106, 743.1236	Theaflavin-3,3′-digallate 3
**6**	8.175/9.296	427.0674/429.1508	C_21_H_16_O_10_	289.0396, 427.0664/ 232.0701	Theaflavic acid
**7**	8.607/-	579.0784/-	C_28_H_20_O_14_	169.0160, 289.0348, 347.8969, 427.0665/-	Epitheaflavic acid 3-gallate/Theaflavic acid 3-gallate
**8**	8.837/-	579.0784/-	C_28_H_20_O_14_	169.0148, 279.0662, 383.0763, 427.0679/-	Epitheaflavic acid 3-gallate/Theaflavic acid 3-gallate
**9**	9.589/-	579.0784/-	C_28_H_20_O_14_	246.0170, 289.0333, 427.0671/-	Epitheaflavic acid 3-gallate/Theaflavic acid 3-gallate
**10**	9.253/9.288	399.0728/401.0851	C_20_H_16_O_9_	165.0179, 233.0451/ 139.0387, 263.0537	Theaflagallin
**11**	11.016/11.024	551.084/553.0959	C_27_H_20_O_13_	169.0139, 381.0616/ 139.0389, 233.0441, 383.0762	Epitheaflagallin 3-O-gallate/Theaflagallin 3-O-gallate

**Table 2:** MS data of major and secondary oxidized products from PPO-catalyzed GCG and C.

Product	Rt (min)	MS (*m*/*z*)	Formula	MS^2^ (*m*/*z*)
Major product **2**	10.359	737 [M+Na−2H]^−^	C_36_H_28_O_16_	125.0236, 429.0576, 599.0818
	10.346	739.1264 [M+Na]^+^	C_36_H_28_O_16_	161.0213, 601.0955
	10.376	717.1449 [M+H]^+^	C_36_H_28_O_16_	139.0390, 277.0704, 409.0905, 579.1130
	10.487	717.1449 [M+H]^+^	C_36_H_28_O_16_	139.0389, 277.0706, 409.0910, 579.1134
Secondary product	10.027	715.1308 [M−H]^−^	C_36_H_28_O_16_	125.0244, 137.0252, 227.0345, 287.0566, 383.0774, 427.0675
	10.001	717.1449 [M+H]^+^	C_36_H_28_O_16_	139.0388, 289.0704, 429.0825, 591.1116

## Data Availability

The original contributions presented in this study are included in the article/Supplementary Material. Further inquiries can be directed to the corresponding authors.

## Supplementary Materials

The following supporting information can be downloaded at: https://www.mdpi.com/article/10.3390/foods14040604/s1, Figure S1: HPLC chromatograms of isoneoTF-3-G and four major theaflavin (non-isomeric) standards. Figure S2: ^13^C-NMR (A) and ^1^H-NMR (B) of major product **2** from PPO-catalyzed GCG and CA. Figure S3: Total ion chromatogram of the MS for major product **2** from PPO-catalyzed GCG and CA. Figure S4: Cytotoxic effects of isoneoTF-3-G, TF-3-G, and 5-FU on HT29 Cells. Table S1: ^13^C-NMR and ^1^H-NMR data of product **2** in CD3OD.

## Abbreviations

BCA, bicinchoninic acid; BCT, black cocoa tea; CA, catechin; *C. ptilophylla*, *Camellia ptilophylla*; CG, catechin gallate; DCFH-DA, 2′,7′-dichlorodihydrofluorescein diacetate; EC, epicatechin; ECG, epicatechin gallate; EDTA, ethylene diamine tetra acetic acid; EGC, epigallocatechin; EGCG, epigallocatechin gallate; ESI, electron spray ionization; ESI-MS, electron spray ionization mass spectrometry; FBS, fetal bovine serum; GC, gallocatechin; GCG, gallocatechin gallate; HPLC, high-performance liquid chromatography; IC_50_, half-inhibitory concentration; isoneoTF-3-G, isoneotheaflavin-3-gallate, isoneotheaflavin 3-O-gallate; MMP, mitochondrial membrane potential; MS, mass spectrometry; MTT, methyl thiazolyl tetrazolium; NMR, nuclear magnetic resonance; PARP, poly(ADPribose) polymerase; PHPLC, preparative high-performance liquid chromatography; PPO, polyphenol oxidase; PVPP, polyvinylpolypyrrolidone; ROS, reactive oxygen species; TBST, Tris-buffered saline tween; TF1, theaflavin; TF-3-G, TF2a, theaflavin-3-gallate, theaflavin 3-O-gallate; TF-3′-G, TF2b, theaflavin-3′-gallate, theaflavin 3′-O-gallate; TF-3,3′-DG, TF3, theaflavin-3,3′-digallate, theaflavin 3,3′-O-digallate; TMS, tetramethyl silane; UPLC-Q-TOF-MS/MS, ultra-high-performance liquid chromatography–quadrupole-time-of-flight tandem mass spectrometry; UV–Vis, ultraviolet–visible; 5-FU, 5-fluorouracil.
