# Supplementary material for: Discovery and Characterization of a Distinctive Theaflavin-3-Gallate Isomer from Camellia ptilophylla with Potent Anticancer Properties Against Human Colorectal Carcinoma Cells
Source: Foods. 2025 Feb 12;14(4):604. doi: 10.3390/foods14040604 (PMC11854890; doi:10.3390/foods14040604)
Supplement: Supplementary file 1 [file foods-14-00604-s001.zip › foods-3372520-supplementary.pdf]

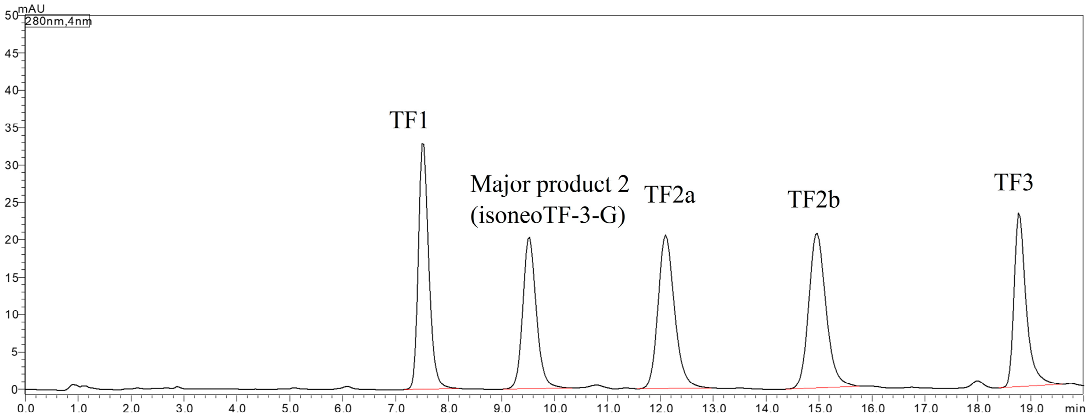


**Figure S1.** HPLC chromatograms of isoneoTF-3-G and four major theaflavin (non-isomeric) standards. Poroshell Ⅱ120 Bonus-RP (4.6×50 mm, 2.7 μm) column; The mobile phase consisted of 0.05% trifluoroacetic acid in ultrapure water (A) and pure acetonitrile (B); The elution was as follows: 0-12 min, 26% B; 12-16 min, 26%-30% B; 16-20 min, 30%-35% B. The column temperature was 30℃ and the flow rate was 0.8 mL/min.

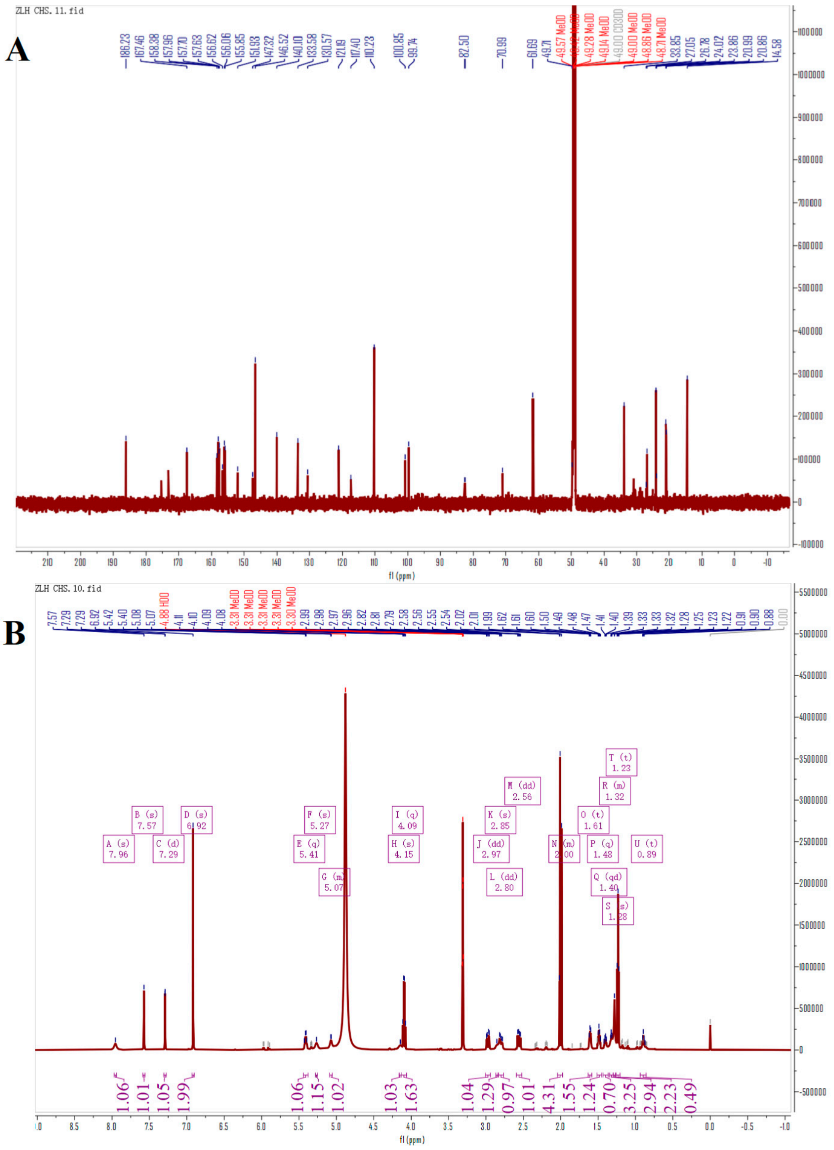


**Figure S2.** $^{13}C$-NMR (A) and $^{1}H$-NMR (B) of major product **2** from PPO-catalyzed GCG and CA.

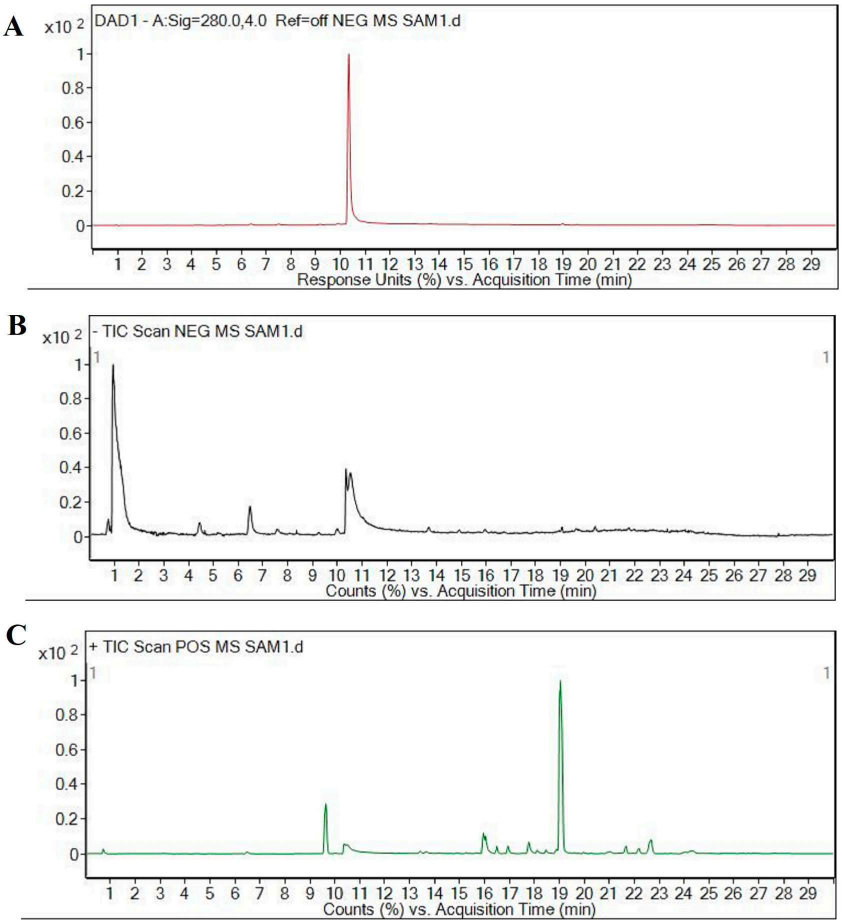


**Figure S3.** Total ion chromatogram of the mass spectrometry for major product **2** from PPO-catalyzed GCG and CA. (A) DAD; (B) Negative ion mode; (C) Positive ion mode.

**Table S1.** $^{13}$C-NMR and $^{1}$H-NMR data of major product **2** in CD3OD

| Position | δ$^{13}$C（ppm） | | δ$^{1}$H（ppm） | |
|---|---|---|---|---|
| | TF-3-G reference values （150 MHz） | Product 2 Observed （151 MHz） | TF-3-G reference values （600 MHz） | Product 2 Observed （600 MHz） |
| C-a (C=O) | 185.6 | 186.23 | | |
| C-G1 (C=O) | 167.4 | 167.46 | | |
| C-9 | 158 | 158.38 | | |
| C-9′ | 157.9 | 157.96 | | |
| C-5 | 157.8 | 157.7 | | |
| C-5′ | 157.3 | 157.63 | | |
| C-7 | 156.4 | 156.62 | | |
| C-7' | 156.3 | 156.06 | | |
| C-c | 155.4 | 155.85 | 7.91 1H s | 7.96 1H s |
| C-h | 151.2 | 151.93 | | |
| C-i | 146.4 | 147.32 | | |
| C-G4, C-G6 | 146.3 | 146.52 | | |
| C-G5 | 139.9 | 140.1 | | |
| C-d | 133.5 | 133.58 | | |
| C-f | 131.3 | 130.57 | | |
| C-G2 | 121 | 121.19 | | |
| C-10 | 99.3 | 99.74 | | |
| C-10′ | 100.2 | 82.5 | | |
| C-e | | | 7.80 1H s | 7.57 1H s |
| C-g | | | 7.38 1H s | 7.29 1H |
| C-G3, C-G7 | | | 6.80 2H s | 6.92 2H s |
| C-6 | | | 6.02 1H d,J=1.8 Hz | |

| | | |
| --- | --- | --- |
| C-6′ | 6.00 1H d,J=2.4 Hz | |
| C-8,C-8′ | 5.99 2H s | |
| C-2 | 5.78 1H m | |
| C-2′ | 5.11 1H s | |
| C-3 | 5.55 1H brs | |
| C-3′ | 4.16 m | |
| | | 5.41 1H q, J=6.5 Hz |
| | | 5.27 1H s |
| | | 5.09-5.05 1H m, J=7.1 Hz |
| | | 4.09 2H q |
| | | 2.97 1H dd, J=16.3 Hz, 5.3 Hz |
| | | 2.8 1H dd，J=16.2 Hz, 7.2 Hz |
| | | 2.56 1H dd, J=15.9 Hz, 8.5 Hz |
| C-4 | 3.07 dd, J=4.8,16.8 Hz; 2.99 dd, J=4.2, 16.8 Hz | 2.04-1.97 4H m |
| | | 1.4 1H qd, J=6.3 Hz, 3.4 Hz |
| | | 1.23 2H t, J=7.1 Hz |
| C-4′ | 2.91 brd, J=16.8 Hz; 2.83 brd, J=16.8 Hz | 1.48 1H q, J=6.0 Hz |

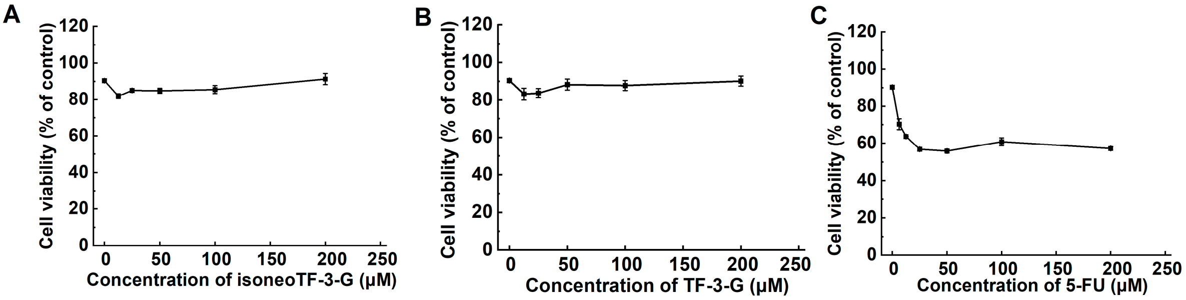


**Figure S4**. Cytotoxic effects of isoneoTF-3-G, TF-3-G, and 5-FU on HT29 Cells. MTT assay was used to determine the cytotoxic effects of isoneoTF-3-G **(A)**, TF-3-G **(B)**, and 5-FU **(C)** on HT29 cells after 48 hours. Viability is expressed as a percentage of the control (100%). A concentration of 0 μM indicates cells treated with the sample solvent only (negative control, cell viability > 90%). 5-FU was used as the positive control. Data are shown as means ± S.D. ($n$ = 3).
